# The FINESSE (Artificial Intelligence Stress Echo) study to develop and validate machine learning–based model to improve risk prediction in patients undergoing stress echocardiography for the assessment of inducible myocardial ischaemia (FINESSE Protocol)

**DOI:** 10.1093/ehjimp/qyag143

**Published:** 2026-08-24

**Authors:** Ugochukwu Ihekwaba, Mohamed Bennasar, Nicholas Johnson, Nerea Sanfeliu Garces, Hanry West, Blaine Price, Jeffrey Khoo, Iain Squire, Kenneth Chan, Attila Kardos

**Affiliations:** Department of Cardiology, Translational Cardiovascular Research Group, Milton Keynes University Hospital NHS Foundation Trust, Milton Keynes, United Kingdom; NIHR Cardiovascular Research Centre, Glenfield Hospital, and Department of Cardiovascular Sciences, University of Leicester, Leicester, United Kingdom; School of Computing and Communications, The Open University, Milton Keynes, United Kingdom; Department of Cardiology, Translational Cardiovascular Research Group, Milton Keynes University Hospital NHS Foundation Trust, Milton Keynes, United Kingdom; Department of Cardiology, Translational Cardiovascular Research Group, Milton Keynes University Hospital NHS Foundation Trust, Milton Keynes, United Kingdom; Division of Cardiovascular Medicine, Radcliffe Department of Medicine University of Oxford, Acute Multidisciplinary Imaging & Interventional Centre, British Heart Foundation, Centre of Research Excellence, Oxford, United Kingdom; School of Computing and Communications, The Open University, Milton Keynes, United Kingdom; NIHR Cardiovascular Research Centre, Glenfield Hospital, and Department of Cardiovascular Sciences, University of Leicester, Leicester, United Kingdom; NIHR Cardiovascular Research Centre, Glenfield Hospital, and Department of Cardiovascular Sciences, University of Leicester, Leicester, United Kingdom; Division of Cardiovascular Medicine, Radcliffe Department of Medicine University of Oxford, Acute Multidisciplinary Imaging & Interventional Centre, British Heart Foundation, Centre of Research Excellence, Oxford, United Kingdom; Department of Cardiology, Translational Cardiovascular Research Group, Milton Keynes University Hospital NHS Foundation Trust, Milton Keynes, United Kingdom; Faculty of Medicine and Health Sciences, University of Buckingham, Buckingham, United Kingdom

**Keywords:** stress echocardiography, artificial intelligence, outcome, MACE, protocol

## Abstract

**Aims:**

Coronary artery disease (CAD) remains a leading cause of morbidity and mortality worldwide, necessitating accurate diagnostic strategies and robust risk stratification. The FINESSE (Artificial Intelligence Stress Echo) study aims to develop and validate machine learning–based models to improve risk prediction in patients undergoing stress echocardiography (SE) for the assessment of inducible myocardial ischaemia, and to implement a structured AI-driven risk reclassification framework to support clinical decision-making.

**Methods and analysis:**

This is a retrospective observational study on prospectively recruited patients referred for SE with suspected CAD. Clinical, demographic, haemodynamic, and echocardiographic data will be extracted from institutional electronic health records and linked with longitudinal national datasets. The study employs a three-stage ML framework: (1) baseline risk estimation using QRISK3, (2) development and optimization of supervised ML models (including ensemble methods, support vector machines, and neural networks), and (3) longitudinal risk prediction using linked NHS England data (such as all-cause mortality and major adverse cardiovascular events, including cardiovascular death, non-fatal myocardial infarction, stroke, unplanned revascularization). Model performance will be evaluated using discrimination (AUC), calibration, and reclassification metrics, and will be externally validated using independent multicentre SE datasets.

**Conclusion:**

The FINESSE study will evaluate the incremental value of machine learning in SE by integrating multimodal data into a clinically actionable risk reclassification framework. This approach has the potential to improve personalized risk stratification, optimize diagnostic pathways, and support precision cardiovascular care.

**Protocol registration:**

Ethical approval has been obtained from the UK Health Research Authority and relevant research ethics committees, with appropriate data governance approvals for the use of routinely collected healthcare data. The FINESSE study is registered on ClinicalTrials.gov (Identifier: NCT07432620), ensuring transparency and public accessibility of the study protocol.

## Graphical Abstract

**Graphical Abstract:**
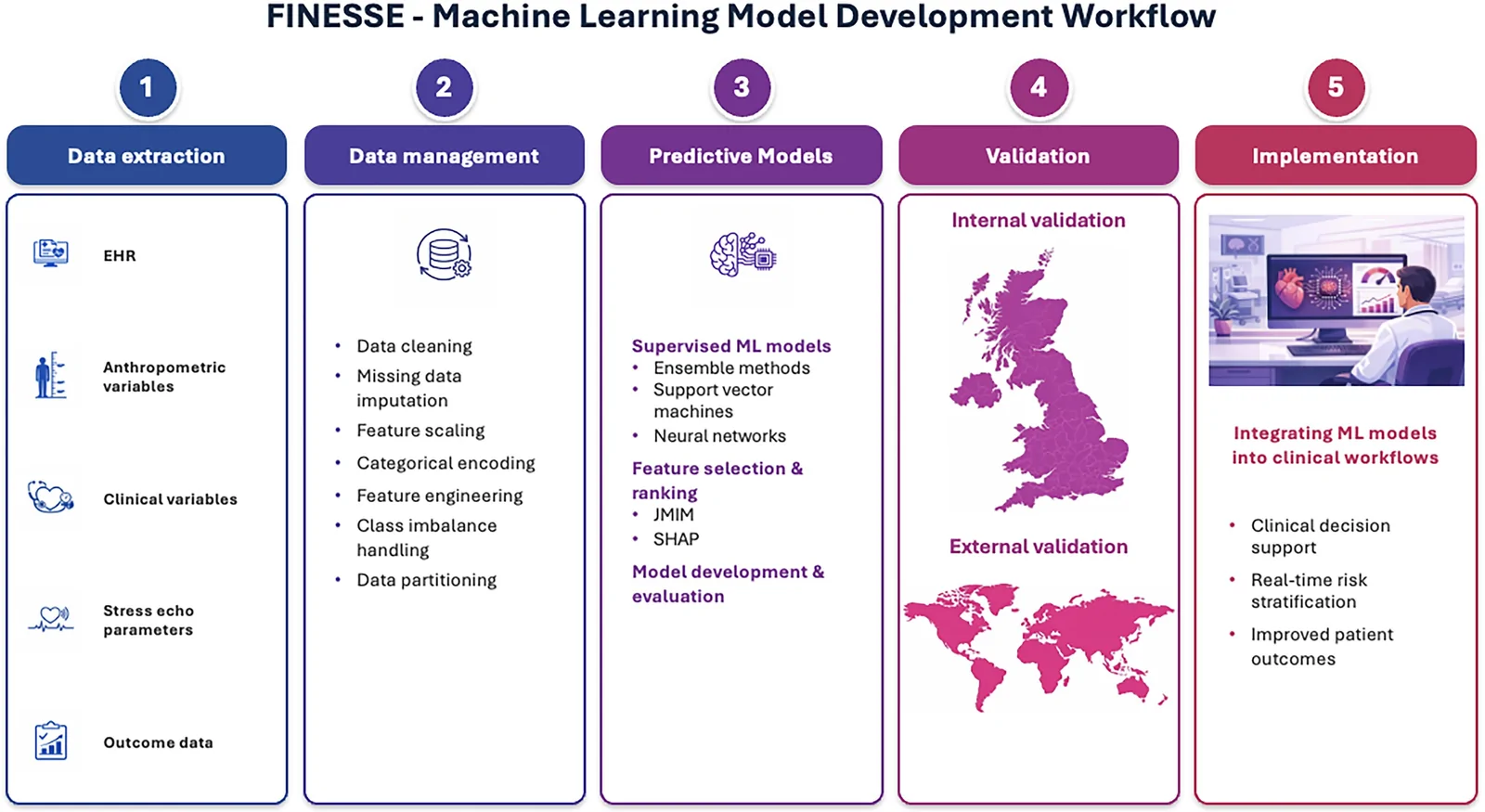

Key messages

- **What is already known on this topic**—Stress echocardiography is a well-established clinical tool in the diagnosis and prognostication of patients with chest pain and effort breathlessness. Machine learning (ML) advances offer an opportunity to improve predictive modelling by integrating diverse clinical, imaging, and demographic datasets
- **What this study adds**—The study seeks to enhance diagnostic accuracy and prognostication compared to traditional methods by integrating echocardiographic parameters with clinical and demographic data.
- **How this study might affect research, practice or policy**—The proposed framework combining ML with clinical information along with SE parameters will be able to help doctors make better decisions about patient management while making healthcare more individualized.

## Introduction

The prevalence of coronary artery disease (CAD) continues to make it one of the top global health issues that cause both disease and death.^1,2^ Effective management relies on precise diagnosis and proper risk stratification. Stress echocardiography (SE) is a non-invasive bedside test used by healthcare professionals to diagnose CAD.^3–6^ It is particularly useful in assessing prognostic disease in patients with suspected CAD.^7,8^ Machine learning (ML) advances offer an opportunity to improve predictive modelling by integrating diverse clinical, imaging, and demographic datasets.^9^ ML algorithms find hidden patterns in data to create better patient risk groups and improve treatment results.^10^ This study aims to leverage ML techniques to develop and validate predictive models for outcomes in patients undergoing SE for the assessment of inducible myocardial ischaemia. The study seeks to enhance diagnostic accuracy and prognostication compared to traditional methods by integrating echocardiographic parameters with clinical and demographic data. Our research will be able to show that combining ML with clinical information along with SE parameters can help doctors make better decisions about patient management while making healthcare more individualized.

## Methods

### Study setting

**Figure 1:**
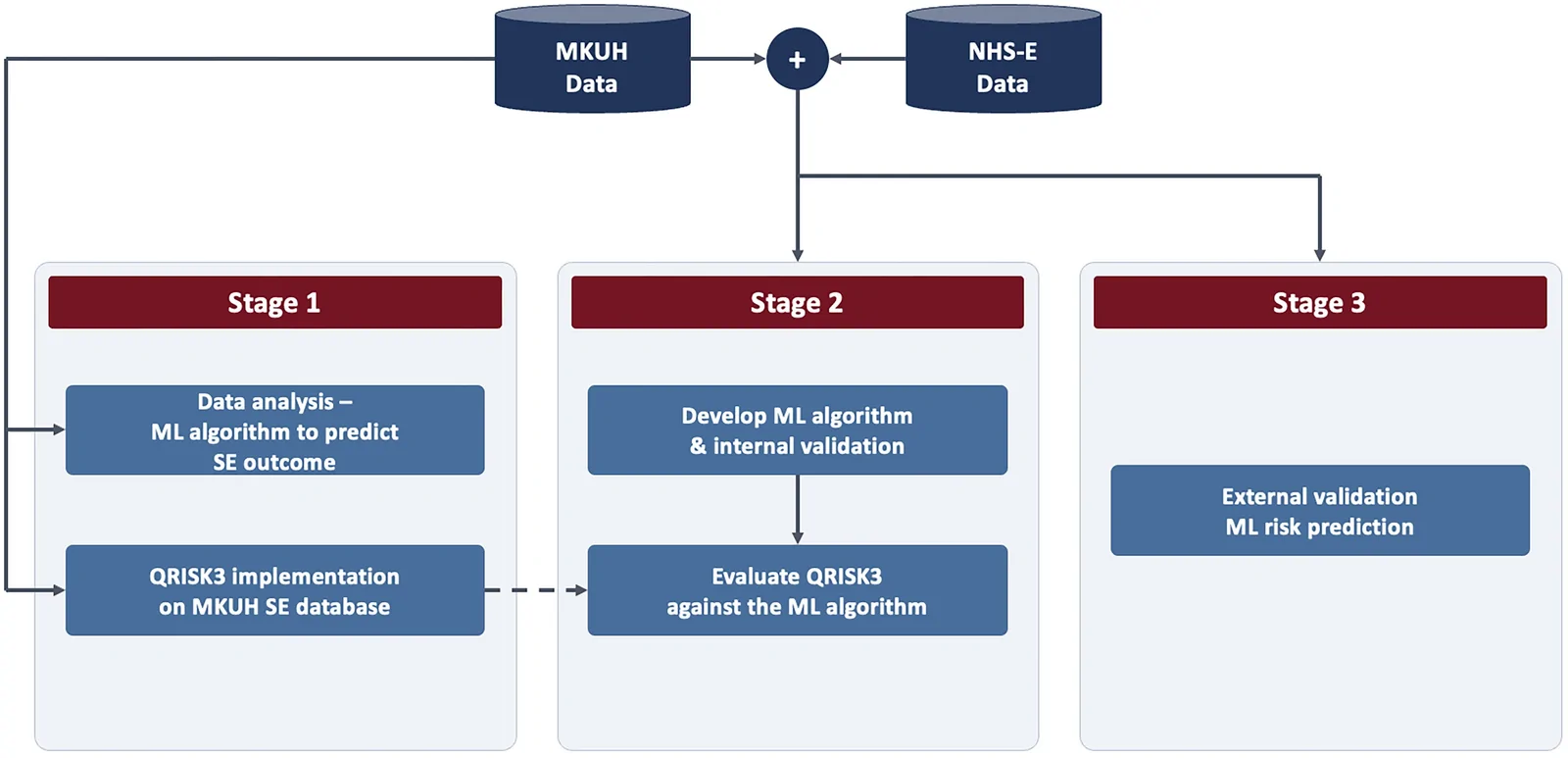
Overview of the three-stage machine learning framework used in the FINESSE study.

This research project examines past data from a single hospital that performed SE on patients suspected of CAD. Our research team retrieved data from the institution's echocardiography and electronic health record (EHR) systems. A standardized data extraction, cleaning, and analysis protocol was implemented to ensure accuracy (*Figure 1*).

### Eligibility criteria

#### Inclusion criteria

The study includes consecutive patients, 18 years or older, referred for a SE for inducible ischaemia detection for suspected CAD.

#### Exclusion criteria

Poor-quality echocardiographic description that preclude accurate interpretation or regional wall motion abnormalities (RWMAs) (thickening) at any stage of the SE protocol. Patients with known allergy to dobutamine or ultrasonic contrast enhancing agents (UCEAs) or who were referred for structural SE to assess severity of valvular abnormalities or left ventricular viability only were excluded. Patients who opted out data sharing were excluded from the study.

### Data collection

Data were collected using both SE reports and institutional EHR databases. Collected patient demographic information will include age, sex, race, height, weight, and body mass index. Cardiovascular risk factors such as; smoking habits, obesity status, family history of CAD, diabetes mellitus, essential hypertension, dyslipidaemia, prior CAD. Patients’ medical history at the time of the SE was also captured. Patients’ variables in support of calculating the QRISK-3 score^11^ was retrieved as complete as data permitted. (QRISK3 data extraction has been described in Supplement document). Extracted echocardiographic parameters include resting LV ejection fraction (from the WMS), and RWMA Wall Motion Score Index (WMSI) of the left ventricular 17 segments at each or the 4 stages of the SE protocol. (Detailed Dobutamine SE protocol and analysis is described in the Supplementary document.) To address inducible ischaemia the difference of the WMSI between low dose and peak stress of Dobutamine SE was calculated.^4,5^ Vital signs including systolic blood pressure (SBP), diastolic blood pressure (DBP), heart rate (HR) at rest and each stage of the SE protocol (low dose, peak stress, and recovery) were collected.

### Outcomes

All-cause death, hard endpoints: cardiovascular death, non-fatal myocardial infarction (MI) and composite outcomes will provide with different major cardiovascular events (MACEs), including stroke and unplanned revascularization, and hospital admission with progressive angina (MACE, 3-4-5) across four time periods: 1–3 years, 3–5 years, 5–10 years, and 10–20 years. The outcome data will be derived from NHS -England database with linking patients’ data such as NHS number and DOB and date of SE over 17 years after the indexed SE. (DARS-NIC-662451-S5L8J-v0.16).

### Sample size calculation

The study aims to include approximately 2300 patients undergoing SE for the assessment of inducible myocardial ischaemia. The sample size was determined based on anticipated event rates, longitudinal outcome modelling requirements, and the complexity of the proposed ML framework.

Previous SE studies and cardiovascular outcome registries suggest that MACE are expected to occur at sufficient frequency within long-term follow-up to support robust prognostic modelling.^12^ The projected cohort size is anticipated to provide an adequate number of outcome events to satisfy event-per-variable requirements for both conventional statistical analyses and ML-based predictive modelling, while reducing the risk of model overfitting. The proposed sample size is also considered sufficient to support internal cross-validation, subgroup analyses, calibration assessment, and the evaluation of multiple supervised ML approaches, including ensemble methods, support vector machines (SVMs), and neural networks. In addition, linkage with longitudinal NHS outcome datasets over extended follow-up periods is expected to strengthen survival modelling and time-to-event prediction analyses.

External validation using independent multicentre SE cohorts is planned to further assess model generalisability and robustness across different clinical settings.

*Table 1* shows the characteristics of the patient population.

**Table 1:** Population characteristics

Variable	Total	Positive SE	Negative SE	*P*-value
Anthropometric variables				
*N*	2263	371 (16%)	1892 (84%)	—
Age (years)	63 (55–72)	64 (57–72)	63 (54–72)	0.031
Male Sex	1201 (53%)	243 (65%)	958 (51%)	<0.0001
Race				
Caucasian	2048 (91%)	330 (89%)	1718 (91%)	0.42
Asian	152 (7%)	27 (7%)	125 (7%)	0.85
Black	39 (2%)	8 (2%)	31 (2%)	0.71
Other	24 (1%)	6 (2%)	18 (1%)	0.31
Height (cm)	167 (160–175)	168 (160–176)	167 (160–175)	0.285
Weight (kg)	80 (68–95)	82 (70–98)	79 (67–94)	0.009
BMI (kg/m^2^)	28.7 (24.8–33.5)	29.0 (25.1–33.9)	28.6 (24.7–33.4)	0.198
BSA (m^2^)	1.90 (1.75–2.08)	1.93 (1.78–2.11)	1.89 (1.74–2.07)	0.012
Cardiovascular risk factors factors				
Hypertension	1371 (61%)	240 (65%)	1131 (60%)	0.072
Dyslipidaemia	1345 (59%)	238 (64%)	1107 (59%)	0.031
Current Smoker	218 (10%)	44 (12%)	174 (9%)	0.092
Former Smoker	648 (29%)	114 (31%)	534 (28%)	0.285
Diabetes Mellitus	671 (30%)	127 (34%)	544 (29%)	0.036
Obesity (BMI ≥30)	232 (10%)	30 (8%)	202 (11%)	0.142
Family History of CAD	996 (44%)	154 (42%)	842 (44%)	NS
Medications				
Beta-blockers	1122 (50%)	216 (58%)	906 (48%)	0.0003
ACE-I/ARB	1205 (53%)	233 (63%)	972 (51%)	<0.0001
Anti-platelets	1346 (59%)	260 (70%)	1086 (57%)	<0.0001
Statins	1320 (58%)	235 (63%)	1085 (57%)	0.028
DSE parameters				
Left Ventricular Ejection				
Fraction (rest)				0.005
Preserved (≥50%)	2153 (95%)	340 (92%)	1813 (96%)	
Mild/Moderate (36–49%)	83 (4%)	24 (7%)	59 (3%)	
Severe (≤35%)	24 (1%)	4 (1%)	20 (1%)	
HR at rest (bpm)	68 (60–77)	65 (58–74)	68 (60–78)	<0.001
HR at peak (bpm)	135 (120–147)	131 (115–144)	136 (122–148)	0.018
SBP rest (mmHg)	138 (125–153)	140 (127–156)	137 (124–152)	0.019
SBP peak (mmHg)	140 (118–160)	135 (112–155)	141 (120–162)	0.0110
DBP rest (mmHg)	80 (70–88)	81 (72–90)	79 (70–87)	0.042
DBP peak (mmHg)	70 (60–80)	68 (58–78)	71 (61–81)	0.011
THR achieved	1650 (73%)	260 (70%)	1390 (73%)	0.21
THR at peak (% of MTHR)	100 (90–110)	98 (85–108)	101 (91–111)	0.045
Double product (mmHg*bpm)	18 564 (15 820–21 330)	17 421 (14 650–20 268)	18 765 (16 093–21 560)	<0.001
Biphasic response (viable-ischaemic)	294 (13%)	59 (16%)	235 (12%)	0.072
WMSI at rest	1.05 (1.00–1.18)	1.12 (1.00–1.35)	1.00 (1.00–1.12)	<0.0001
WMSI at peak	1.06 (1.00–1.24)	1.18 (1.05–1.41)	1.00 (1.00–1.18)	<0.0001
Delta WMSI	0.00 (0.00–0.12)	0.05 (0.00–0.18)	0.00 (0.00–0.05)	<0.0001
Positive DSE (inducible ischaemia)	371 (16%)	371 (100%)	0 (0%)	—
Number of inducible ischaemic segments				
0	1896 (84%)	0 (0%)	1896 (100%)	—
1–2	250 (11%)	250 (67%)	0	—
3–4	100 (4%)	100 (27%)	0	—
≥5	21 (1%)	21 (6%)	0	—
QRISK3 score	8.4 (3.4–15.2)	9.6 (4.6–18.2)	8.1 (3.2–14.4)	0.008

Data are: Median (25–75 percentiles) for continuous variables, or *n* (%) for categorical variables. Left ventricular ejection fraction percentages are based on patients with categorized data (*n* = 2260; 21 missing/uncategorizable). Race approximated from ethnicity categories. Comparison by Mann–Whitney test for continuous variables and Chi-square test for categorical variables (for multi-category, overall test). *P* < 0.05 indicates statistical significance.

### Machine learning framework

The proposed ML framework comprises three sequential stages, as illustrated in *Figure 1*. Stage 1 focuses on data preprocessing and baseline risk estimation using the QRISK3 clinical risk calculator. Stage 2 applies supervised ML models to predict disease outcomes and stratify patient risk. Stage 3 integrates longitudinal NHS Digital data with the local Hospital cohort to forecast future cardiovascular events.

### Stage 1: clinical risk modelling and baseline assessment

#### QRISK3 risk estimation

QRISK3 is a validated clinical risk prediction model designed to estimate the 10-year risk of cardiovascular disease (CVD) using demographic and clinical variables.^11^ In this study, QRISK3 is used as a benchmark model against which ML-based approaches are evaluated.

Relevant patient characteristics and CAD risk factors were extracted from the local Hospital dataset and processed using the QRISK3 framework. The resulting risk estimates provide a clinically interpretable baseline for comparison with ML predictions (details of data extraction is provided in the Supplement).

#### Data sources and extraction

Data were obtained from institutional EHR and SE databases, including demographic information, medical history, laboratory measurements, medication records, and imaging-derived parameters. During data extraction, quality control procedures were applied to identify missing values, inconsistencies, and outliers. All data processing and cleaning were performed using Python-based data analysis tools. Extracted variables were structured into a unified tabular format suitable for statistical analysis and ML modelling.

#### Baseline performance evaluation

Baseline predictive performance will be established using QRISK3 risk estimates. Model performance will be assessed using accuracy, sensitivity, specificity, and area under the receiver operating characteristic curve (AUC, ROC). Calibration analysis and ROC curves will be used to compare predicted risk with observed outcomes. These results serve as a reference for assessing the added value of ML-based models.^13–18^

#### Data preprocessing

Prior to ML modelling, the dataset will undergo a structured preprocessing pipeline to ensure data quality and analytical robustness.

1. Data cleaning: Duplicate records, implausible values, and inconsistencies were identified and corrected where appropriate. Outliers were assessed using statistical and clinically informed thresholds.
2. Missing data handling: The extent and pattern of missing data are assessed prior to analysis. Continuous variables are imputed using median, k-nearest neighbour (KNN), or multiple imputation methods, as appropriate, while categorical variables are imputed using mode-based methods. To minimize information leakage, imputation is performed using the training dataset only, with the learned imputation parameters subsequently applied to the validation dataset. No clinically implausible default values were assigned during preprocessing.
3. Feature scaling: Continuous variables were standardized or normalized to ensure numerical stability and compatibility across ML algorithms.
4. Categorical encoding: Categorical variables (e.g. sex, smoking status, ethnicity) were converted into numerical representations using appropriate encoding techniques.
5. Feature engineering: Additional clinically relevant variables, including stress–response and haemodynamic indices, were be derived from existing measurements.
6. Class imbalance handling: Outcome imbalance will be assessed and addressed using strategies such as class weighting or oversampling within the training dataset.
7. Data partitioning: The dataset is randomly divided into development (80%), internal validation (20%) cohorts using stratified sampling to preserve event rates across all datasets. Hyperparameter optimization is restricted to the development cohort with internal validation performed exclusively within the validation dataset.

### Stage 2: machine learning model development

The cohort is divided into training and validation sets, with 20% of the data held out for internal validation. Supervised machine learning models are developed using demographic characteristics, clinical risk factors, physiological measurements obtained during SE, and imaging-derived variables. The evaluated algorithms include ensemble methods (Random Forest and Gradient Boosting), SVM, and artificial neural networks (ANNs). Hyperparameter optimizations are performed using Bayesian optimization within nested cross-validation. Model selection is based on discrimination, calibration, robustness across validation folds, and clinical interpretability. SHapley Additive exPlanations (SHAP) is subsequently used to quantify feature importance and improve model transparency.

#### Feature selection and ranking

Feature importance will be assessed using two complementary approaches:

1. **Joint mutual information maximization (JMIM)** ^19^: JMIM is a filter-based feature selection method that evaluates the joint dependency between candidate features and the outcome variable while accounting for redundancy among selected features. As a classifier-independent approach, JMIM is well suited for identifying informative features in high-dimensional clinical data.
2. **SHapley Additive exPlanations (SHAP)** ^20^: SHAP values will be computed to quantify the contribution of individual features to model predictions, supporting interpretability and facilitating clinical insight into model behaviour.

#### Model development and evaluation

Selected features will then be used for prognostic modelling using approached such as logistic regression, LASSO regression (Least Absolute Shrinkage and Selection Operator), Cox survival model, and Random Forest survival model. Hyperparameters will be optimized using grid search and random search strategies. Model performance will be evaluated using accuracy, sensitivity, specificity, true positive rate, and true negative rate. A five-fold cross-validation strategy will be employed to ensure robustness and reduce sampling bias. In each iteration, models will be trained on four folds and will be evaluated on the remaining fold, with the process repeated until all folds were used for validation.

To minimize overfitting, model development incorporates a held-out validation cohort for internal performance assessment, and final model performance will be further evaluated in geographically distinct external validation cohorts with long-term clinical follow-up (including EVAREST and Stress Echo 2030, where available) to assess generalisability.

### Stage 3: model evaluation, longitudinal risk prediction using NHS England (aka digital) data

In the final stage, the local Hospital dataset was linked with NHS England longitudinal records, which include confirmed cardiovascular events. The performance of ML score to predict clinical events will be evaluated against base models of QRISK3, and SE ischaemia positivity. The forecasting power of future cardiovascular events over 5-year and 10-year will be assessed.

This longitudinal modelling framework enables dynamic risk prediction and supports early identification of individuals at elevated risk of adverse cardiovascular outcomes. We will be inviting external sites for validation and testing the robustness and scalability of the developed predictive algorithm in all spectra of SE modalities.

Internal validation once completed external validation is performed using independent multicentre cohorts, including EVAREST and Stress Echo 2030 to assess generalisability of the model. Model discrimination will be assessed using the area under the receiver operating characteristic curve (AUC) with 95% confidence intervals (CIs). Calibration will be evaluated using calibration-in-the-large, calibration slope, observed-to-expected event ratios, calibration plots, and the Brier score. Where systematic miscalibration is identified, model recalibration using intercept and slope adjustment will be undertaken prior to assessing clinical utility by. using risk reclassification analyses, including the net reclassification improvement (NRI).

### Statistical methods

Participant demographics and characteristics are summarized with descriptive statistics. Categorical variables are expressed as median (interquartile range), and continuous variables are expressed as mean (standard deviation). Internal model validation will be performed with 5-fold cross-validation. Cox-regression model was fitted to estimate the hazard rates, hazard ratios (HR) and the 95% CIs. Survival probabilities from any event for the first time since SE will be evaluated using Kaplan–Meier curves. Performance evaluation for the model will include measurements of Accuracy alongside Sensitivity and Specificity, using receiver Operating Characteristic (ROC) analysis with c-statistics, together with calibration plots and risk reclassification. The study will perform comparative assessments between ML models and the QRISK3 calculator to evaluate their incremental predictive value. Statistical analyses will be performed using Stata 18.0 (StataCorp LP, College Station, TX) and the R environment (R 4.5.1, The R Foundation for Statistical Computing, http://www.R-project.org) using R studio (version 2024.12.1). All tests were two-sided and α was set at 0.05, unless specified otherwise.

### FINESSE AI–based risk reclassification framework (*Figure 2*)

To extend the predictive modelling framework described above, this study will implement a structured risk reclassification approach using FINESSE AI. This component is designed to translate model-derived risk estimates into clinically actionable categories and integrate them within existing diagnostic and management pathways.

**Figure 2:**
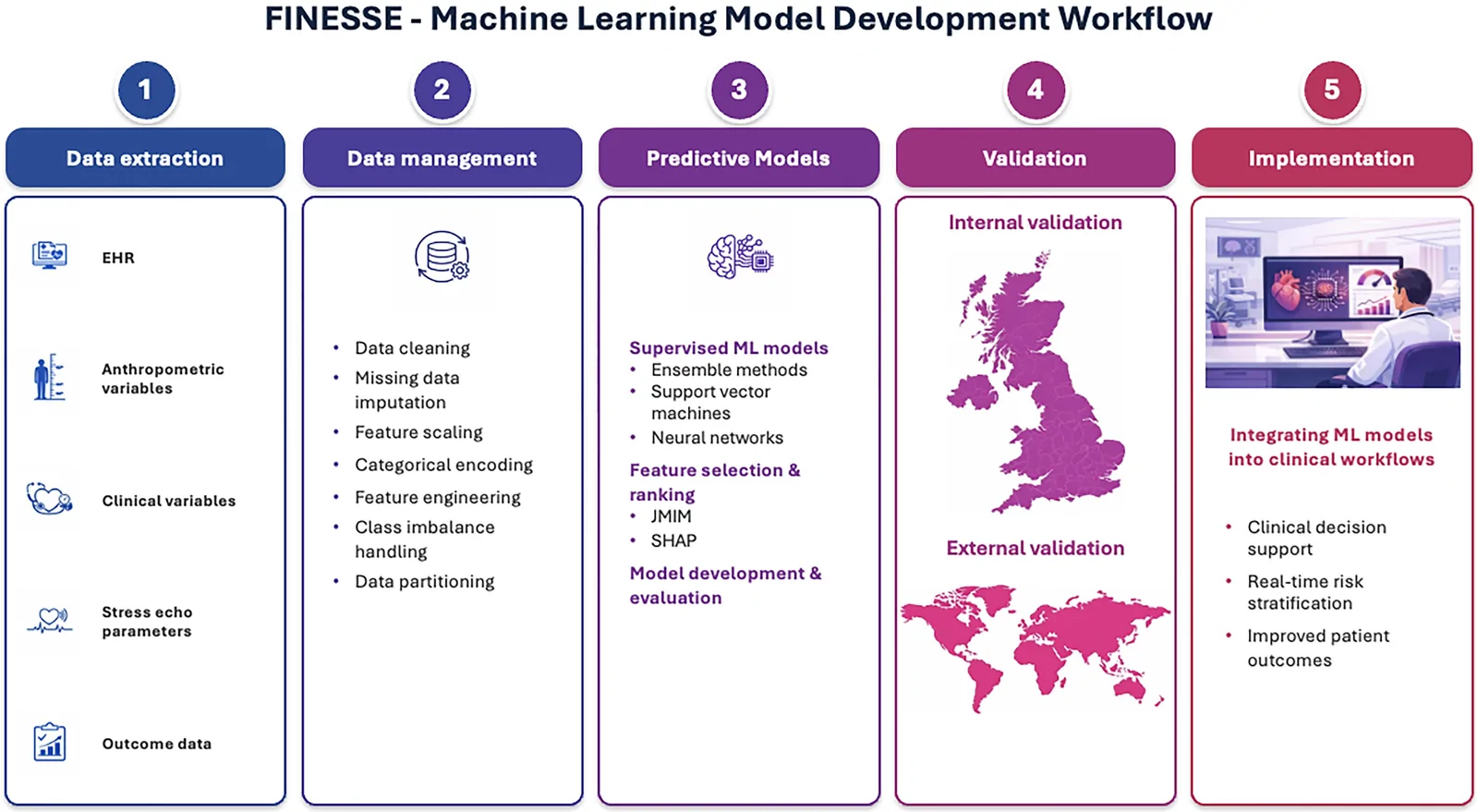
FINESSE—machine learning model development workflow.

Baseline risk for each patient will be defined using conventional clinical assessment and QRISK3 derived estimates, as previously described. FINESSE AI will then generate an updated, individualized probability of the outcome of interest at a specified time horizon by integrating echocardiographic parameters, haemodynamic response variables, clinical characteristics, and longitudinal data. The integrated risk estimate will be defined as:


$$R_{F}(i,t)=f(R_{0}(i,t),X_{e}(i),X_{h}(i),X_{c}(i),X_{l}(i))$$

where $R_{0}(i,t)$ represents the baseline risk estimate and the additional inputs correspond to echocardiographic, haemodynamic, clinical, and longitudinal variables, respectively.

To facilitate clinical interpretation, continuous risk estimates will be mapped to predefined categories using a threshold-based classification function. These thresholds will be prespecified and subsequently evaluated against observed event rates within the study cohort to ensure appropriate calibration defined as low risk–intermediate risk–high risk.

Risk reclassification will be defined as a change in category between baseline and FINESSE-derived estimates, expressed as the difference between the categorical assignments derived from $R_{F}(i,\;t)$ and $R_{0}(i,\;t)$. Reclassification will therefore reflect either upward or downward movement across predefined risk strata. To minimize clinically insignificant shifts, reclassification will only be considered when the absolute change in predicted risk exceeds a predefined threshold, anticipated to be in the range of 3–5 percentage points. The predefined 3–5% risk reclassification threshold was selected because it represents the range within which relatively small improvements in estimated cardiovascular risk may influence guideline-directed management decisions, including initiation or intensification of lipid-lowering therapy, additional diagnostic testing, or referral for invasive coronary angiography. Similar thresholds have been adopted in previous cardiovascular risk prediction studies evaluating improvements in discrimination and clinical utility through net reclassification analyses. The threshold was therefore chosen to maximize clinical relevance rather than statistical optimization alone.

In instances where FINESSE-derived risk estimates are discordant with conventional diagnostic findings, such as a low-risk SE profile in the presence of an elevated predicted risk, cases will be flagged for clinical review rather than automatically reassigned. This approach is intended to preserve alignment with established diagnostic pathways and ensure that model outputs are interpreted within the broader clinical context.

Rather than replacing current clinical assessment, the FINESSE framework is intended to function as a decision-support system embedded within routine SE reporting. Following completion of image interpretation, the algorithm automatically integrates demographic, clinical, laboratory, ECG and echocardiographic parameters to generate an individualized risk estimate. This risk estimate may assist clinicians in identifying patients who would benefit from intensified preventive therapy, further anatomical imaging, closer surveillance, or expedited invasive investigation while preserving final clinical judgement.

The incremental value of the FINESSE AI reclassification framework will be evaluated by comparing baseline and model-derived predictions using discrimination metrics, including the AUC, alongside calibration assessment using calibration plots and Brier scores. In addition, reclassification-specific metrics, including NRI and integrated discrimination improvement, will be used to quantify the extent to which FINESSE AI enhances patient stratification beyond conventional approaches.

### Ethics and dissemination

This study is conducted in accordance with the principles outlined in the *Declaration of Helsinki* and complies with all relevant UK regulatory and governance frameworks for health research. Ethical approval for the FINESSE project was obtained through the UK Health Research Authority (HRA) via the Integrated Research Application System (IRAS). The study received a favourable ethical opinion from the Yorkshire & The Humber—South Yorkshire Research Ethics Committee (REC) under REC reference: 19/YH/0159, with IRAS project ID: 249297. Subsequent protocol amendments, including version 6.1 (26 January 2022), were reviewed and approved with a favourable opinion by the same REC. All study procedures were therefore authorized to proceed in line with REC governance requirements.

In addition to REC approval, the study underwent review through the HRA approval process, ensuring compliance with NHS research governance, legal, and operational requirements across participating sites. Local approval was granted by the Milton Keynes University Hospital NHS Foundation Trust Research & Development (R&D) department (Protocol Record: MKUH-RD-008, Artificial Intelligence Stress Echo [FINESSE] Project).

Given the nature of the study, involving the use of routinely collected healthcare data and linkage with national datasets, additional regulatory approvals were required under UK data protection legislation. Approval for the use of confidential patient information without explicit consent was obtained from the Confidentiality Advisory Group (CAG) under HRA reference: 22/CAG/0034, following Section 251 of the NHS Act 2006. This approval permits the lawful processing and linkage of identifiable data where obtaining individual consent is not feasible, subject to strict safeguards.

Data linkage was conducted through secure NHS England data environments, following established governance pathways (DARS-NIC-662451-S5L8J-v0.16). Patient-level data from local NHS systems are pseudonymized prior to transfer and linkage with national datasets (e.g. mortality and hospital episode statistics). All data processing complies with the UK General Data Protection Regulation (UK GDPR) and the Data Protection Act 2018, with strict controls on access, storage, and data minimization. Only authorized personnel have access to de-identified datasets for analysis.

The FINESSE study is registered on ClinicalTrials.gov (Identifier: NCT07432620), ensuring transparency and public accessibility of the study protocol. Dissemination of results will occur through peer-reviewed publications, conference presentations, and engagement with clinical and academic stakeholders. Findings will be reported in accordance with relevant reporting guidelines (e.g. TRIPOD), ensuring reproducibility and transparency.

No individual participants will be identifiable in any dissemination outputs. Data sharing will follow institutional and regulatory policies, and any secondary use of data will require appropriate approvals.

### Confidentiality and access to data

The research team will anonymize patient data before securely storing it on institutional servers. Access to the dataset will be restricted to authorized research personnel only. Collaborative studies will operate under established data-sharing agreements that meet institutional and regulatory standards.

### Participant safety

This study is a retrospective observational analysis using routinely collected healthcare data and therefore poses minimal risk to participants. No direct patient contact, intervention, or alteration to clinical care is involved.

A waiver of informed consent was granted on the basis that the study uses existing data, involves no additional risk to participants, and that obtaining consent would be impracticable given the scale and retrospective nature of the dataset. The use of confidential patient information without explicit consent is supported by regulatory approvals under Section 251, with appropriate safeguards in place.

The primary potential risk relates to data confidentiality. This is mitigated through a structured data governance framework in which data are pseudonymized at source prior to transfer for research purposes. Identifiable information is not accessible to the research team. Data linkage with national datasets is performed within secure NHS data environments, using established processes that ensure separation of identifiers from clinical data. Access to data is restricted to authorized personnel only, with role-based permissions, secure storage, and full audit trails in place. All analyses are conducted on de-identified datasets, and results are reported in aggregate form to prevent re-identification of individuals. Given these safeguards and the non-interventional study design, no physical or psychological risks to participants are anticipated.

### Dissemination policy

The research findings will reach their audience through publications in peer-reviewed journals alongside conference presentations and institutional seminars. Clinicians will receive data-driven insights to support their decision-making processes in SE.

### Patient and public involvement

Patients and members of the public were not involved in the initial design of this retrospective study, as it is based on the analysis of pre-existing routinely collected clinical data. However, patient and public involvement (PPI) has been incorporated into the governance and oversight of the project. A dedicated PPI group contributed to the review and monitoring of the study’s data governance processes, particularly in relation to the use of confidential patient information without consent. This group provided input during the Confidentiality Advisory Group (CAG) approval process and has continued to offer oversight through regular follow-up and review meetings. Their role included ensuring that the use of patient data remained proportionate, transparent, and aligned with public expectations, as well as advising on communication and acceptability of data use.

While patients were not directly involved in defining the analytical methodology, their involvement has informed the ethical acceptability and accountability of the research.

This study represents an important step toward the responsible integration of machine learning into clinical practice, with ongoing commitment to strengthening patient-centred approaches in subsequent research phases.

### Expected outcomes

Development of a robust ML-based predictive model for adverse outcomes in SE. Identification of significant predictors, including echocardiographic, clinical, and demographic variables. Demonstration of the incremental value of ML over traditional statistical methods in improving diagnostic accuracy and risk stratification (*Figure 2*). Insight into the feasibility of integrating ML models into clinical workflows to personalize patient care and optimize resource utilization. (*Figure 2*).

### Patients characteristics & discussion

Quantitative machine learning performance analyses, including comparative AUC, NRI, integrated discrimination improvement, and calibration slope, will be reported in the primary results manuscript and have therefore been intentionally omitted from this protocol paper.

From the 2281 patients 14 patients’ data were excluded from analysis due to their indication for severity of aortic valve stenosis and viability only assessment in 8 and 6 patients respectively. As summarized in *Table 1*, of the 2267 patients undergoing dobutamine SE the median age was 63 years, 53% male, predominantly Caucasian race. Sixty-one percent had the diagnosis of hypertension, 59% dyslipidaemia, 30% diabetes mellitus, 10% current and 29% former smokers, and 10% obese (BMI≥30). The echocardiographic variables indicated 95% of the patients had preserved LVEF. During dobutamine SE 73% of patients achieved target HR (defined as 85% of the age predicted maximum HR), 16% had inducible myocardial wall motion abnormality, and 13% had biphasic response in keeping with viable and ischaemic response. Based on the number of inducible ischaemia of the 17 LV segments low-risk results (no inducible ischaemia) were present in 84%, intermediate-risk results (1–2 new RWMA) in 11%, and high-risk results (≥3 new RWMA) in 5% of cases. Patients with positive Dobutamine SE were more likely to be older males with traditional cardiovascular risk factors, particularly dyslipidaemia (64% vs. 59%, *P* = 0.031) and diabetes mellitus (34% vs. 29%, *P* = 0.036). They exhibited a higher burden of established disease, reflected by greater use of beta-blockers, ACE-I/ARB, anti-platelets, and nitrates, as well as higher QRISK3 scores (median 9.6 vs. 8. 1, *P* < 0.008). These findings align with established literature showing that male sex, dyslipidaemia, diabetes, and the use of cardiovascular medication predict inducible ischaemia on stress testing.^7,8^

Left ventricular systolic function was more frequently impaired in the positive group, with mild-to-moderate dysfunction (LVEF 36–49%) occurring in 7% vs. 3% (*P* = 0.005). During SE positive patients showed blunted chronotropic response (lower peak HR *P* = 0.018), albeit with no difference of the achieved target HR (70% vs. 73%, *P* = 0.21), consistent with higher beta-blocker use and possible underlying autonomic or myocardial dysfunction. The independent prognostic value of chronotropic incompetence during different forms of SE has recently been reported.^21,22^ As expected, peak and delta WMSI were significantly higher in the positive group.

### Novelty and technical contribution of the FINESSE AI framework (*Figure 2*)

The FINESSE framework extends beyond conventional ML applications in cardiovascular risk prediction by integrating multimodal clinical, haemodynamic, echocardiographic, and longitudinal outcome data within a unified AI-driven decision-support framework. Unlike traditional risk models that rely primarily on static demographic and clinical variables, FINESSE incorporates dynamic stress–response features derived during SE, including temporal haemodynamic changes and WMSI trajectories across different stages of the stress protocol.

A key innovation of the framework lies in the integration of patients anthropometric and, clinical variables, SE data with long-term NHS-linked outcome datasets, enabling longitudinal modelling of cardiovascular risk over extended follow-up periods. In addition, the proposed framework incorporates AI methodologies, including JMIM and SHAP, to improve interpretability, identify clinically relevant predictors, and support transparency in model decision-making. Rather than functioning solely as a prediction tool, FINESSE introduces an AI-driven risk reclassification framework designed to generate updated individualized risk estimates and translate them into clinically actionable risk categories aligned with contemporary diagnostic and management pathways.

External validation (using established International, multicentre and UK-based National SE databases) will strengthen the reliability and applicability of the findings in diverse clinical settings. Limitations include its retrospective design, potential for selection bias, and reliance on data quality from EHRs. Future research should focus on prospective studies, real-world implementation, and the development of user-friendly interfaces for clinicians.

## Supplementary Material

qyag143_Supplementary_Data

## Data Availability

The data underlying this article will be shared on reasonable request to the corresponding author.

## Lead Author Biography

Dr Ugochukwu Kelechi Ihekwaba is a Consultant Cardiologist at Milton Keynes University Hospital NHS Foundation Trust and Oxford University Hospitals NHS Foundation Trust. He graduated from the University of Sheffield with degrees in Biochemistry and Medicine and holds accreditation in transthoracic echocardiography from the British Society of Echocardiography, as well as certification as a Cardiac Device Specialist from the International Board of Heart Rhythm Examiners. His clinical interests include cardiovascular imaging, cardiac devices, coronary artery disease, and heart failure. His research focuses on stress echocardiography, cardiovascular outcomes, machine learning applications in cardiology, and predictive modelling. He has led multiple systematic reviews and meta-analyses in cardiovascular imaging and is the principal investigator of the FINESSE project, which aims to integrate clinical, imaging, and artificial intelligence approaches to improve cardiovascular risk prediction and patient management.

## References

[1] Sweeney M, Bleeze G, Storey S, Cairns A, Taylor A, Holmes C et al The impact of an acute chest pain pathway on the investigation and management of cardiac chest pain. Future Healthc J 2020;7:53–9. 32104767 10.7861/fhj.2019-0025 PMC7032589

[2] Goodacre S, Cross E, Arnold J, Angelini K, Capewell S, Nicholl J. The health care burden of acute chest pain. Heart (British Cardiac Society) 2005;91:229–30. 15657244 10.1136/hrt.2003.027599 PMC1768669

[3] Vrints C, Andreotti F, Koskinas KC, Rossello X, Adamo M, Ainslie J et al 2024 ESC guidelines for the management of chronic coronary syndromes. Eur Heart J 2024;45:3415–537.Erratum in: Eur Heart J. 2025 Feb 21:ehaf079. doi: 10.1093/eurheartj/ehaf079. 39210710 10.1093/eurheartj/ehae177

[4] Pellikka PA, Arruda-Olson A, Chaudhry FA, Chen MH, Marshall JE, Porter TR et al Guidelines for performance, interpretation, and application of stress echocardiography in ischemic heart disease: from the American society of echocardiography. J Am Soc Echocardiogr 2020;33:1–41.e8. 31740370 10.1016/j.echo.2019.07.001

[5] Picano E, Pierard L, Peteiro J, Djordjevic-Dikic A, Sade LE, Cortigiani L et al The clinical use of stress echocardiography in chronic coronary syndromes and beyond coronary artery disease: a clinical consensus statement from the European association of cardiovascular imaging of the ESC. Eur Heart J Cardiovasc Imaging 2024;25:e65–90. 37798126 10.1093/ehjci/jead250

[6] Marwick TH . Stress echocardiography. Heart 2003;89:113–8. 12482809 10.1136/heart.89.1.113 PMC1767520

[7] Woodward W, Johnson CL, Krasner S, O’Driscoll J, McCourt A, Dockerill C et al Long-term outcomes after stress echocardiography in real-world practice: a 5-year follow-up of the UK EVAREST study. Eur Heart J Cardiovasc Imaging 2025;26:187–96. 39531637 10.1093/ehjci/jeae291 PMC11781832

[8] Ihekwaba U, Johnson N, Choi JS, Savarese G, Orsini N, Khoo J et al Long-term prognostic value of contemporary stress echocardiography in patients with suspected or known coronary artery disease: systematic review and meta-analysis. Heart 2024;110:1349–56. 39179369 10.1136/heartjnl-2024-324534 PMC11671965

[9] Motwani M, Dey D, Berman DS, Germano G, Achenbach S, Al-Mallah MH et al Machine learning for prediction of all-cause mortality in patients with suspected coronary artery disease: a 5-year multicentre prospective registry analysis. Eur Heart J 2017;38:500–7. 27252451 10.1093/eurheartj/ehw188 PMC5897836

[10] Montomoli J, Romeo L, Moccia S, Bernardini M, Migliorelli L, Berardini D et al Machine learning using the extreme gradient boosting (XGBoost) algorithm predicts 5-day delta of SOFA score at ICU admission in COVID-19 patients. J Intensive Med 2021;1:110–6. 36785563 10.1016/j.jointm.2021.09.002 PMC8531027

[11] Hippisley-Cox J, Coupland C, Brindle P. Development and validation of QRISK3 risk prediction algorithms to estimate future risk of cardiovascular disease: prospective cohort study. BMJ 2017;357:j2099. 28536104 10.1136/bmj.j2099 PMC5441081

[12] Cortigiani L, Azzolina D, Ciampi Q, Lorenzoni G, Gaibazzi N, Rigo F et al Machine learning algorithms for prediction of survival by stress echocardiography in chronic coronary syndromes. J Pers Med 2022 Sep 16;12:1523. 36143307 10.3390/jpm12091523 PMC9504503

[13] Huang M-W, Tsai C-F, Tsui S-C, Lin W-C. Combining data discretization and missing value imputation for incomplete medical datasets. PLoS One 2023;18:e0295032. 38033140 10.1371/journal.pone.0295032 PMC10688879

[14] Chua AE, Pfeifer LD, Sekera ER, Hummon AB, Desaire H. Workflow for evaluating normalization tools for omics data using supervised and unsupervised machine learning. J Am Soc Mass Spectrom 2023;34:2775–84. 37897440 10.1021/jasms.3c00295 PMC10919320

[15] Kumar V, Lalotra GS, Sasikala P, Rajput DS, Kaluri R, Lakshmanna K et al Addressing binary classification over class imbalanced clinical datasets using computationally intelligent techniques. Healthcare 2022;10:1293. 35885819 10.3390/healthcare10071293 PMC9322725

[16] Noroozi Z, Orooji A, Erfannia L. Analyzing the impact of feature selection methods on machine learning algorithms for heart disease prediction. Sci Rep 2023;13:22588. 38114600 10.1038/s41598-023-49962-w PMC10730875

[17] Ozcift A, Gulten A. Classifier ensemble construction with rotation forest to improve medical diagnosis performance of machine learning algorithms. Comput Methods Programs Biomed 2011;104:443–51. 21531475 10.1016/j.cmpb.2011.03.018

[18] Miao K, Miao J, Miao G. Diagnosing coronary heart disease using ensemble machine learning. Int J Adv Comput Sci Appl 2016;7.

[19] Bennasar M, Hicks Y, Setchi R. Feature selection using joint mutual information maximisation. Expert Syst Appl 2015;42:8520–32.

[20] Lundberg SM, Lee SI. A unified approach to interpreting model predictions. Adv Neural Inf Process Syst 2017;30:4768–77.

[21] Cortigiani L, Ciampi Q, Zagatina A, Kalinina E, Padang R, Kane GC et al Chronotropic incompetence during exercise or pharmacological stress is associated with reduced survival in patients with chronic coronary syndromes. Eur J Prev Cardiol 2025 Sep 5:zwaf492. 40908497 10.1093/eurjpc/zwaf492

[22] Kardos A, Johnson C, Leeson P. Chronotropic incompetency during stress echocardiography: a new paradigm for mortality and cardiac event prediction? Eur J Prev Cardiol 2025 Oct 9:zwaf550. 41065274 10.1093/eurjpc/zwaf550

