# Supplementary material for: The FINESSE (Artificial Intelligence Stress Echo) study to develop and validate machine learning–based model to improve risk prediction in patients undergoing stress echocardiography for the assessment of inducible myocardial ischaemia (FINESSE Protocol)
Source: Eur Heart J Imaging Methods Pract. 2026 Aug 24;4(3):qyag143. doi: 10.1093/ehjimp/qyag143 (PMC13541104; doi:10.1093/ehjimp/qyag143)
Supplement: Supplementary file 1 [file qyag143_supplementary_data.docx]

**Supplementary documents**

The FINESSE (Artificial Intelligence Stress Echo) study to develop and validate machine learning–based model to improve risk prediction in patients undergoing stress echocardiography for suspected CAD**. (**FINESSE Protocol)

**Authors:**

Ugochukwu Ihekwaba^1,2^, Mohamed Bennasar^3^, Nicholas Johnson^1^, Nerea Sanfeliu Garces^1^, Hanry West^4^, Blaine Price^3^, Jeffery Khoo ^2^ Iain Squire ^2^ Kenneth Chan^4^, Attila Kardos^1,5^

**Institutions**

1.Department of Cardiology, Translational Cardiovascular Research Group, Milton Keynes University Hospital NHS Foundation Trust, Milton Keynes,

2.NIHR Cardiovascular Research Centre, Glenfield Hospital, and Department of Cardiovascular Sciences, University of Leicester, Leicester, United Kingdom

3. School of Computing and Communications, The Open University, Milton Keynes, United Kingdom

4. Acute Multidisciplinary Imaging & Interventional Centre, British Heart Foundation

Centre of Research Excellence, Division of Cardiovascular Medicine, Radcliffe Department of Medicine university of Oxford, Oxford, United Kingdom

5. Faculty of Medicine and Health Sciences, University of Buckingham, Buckingham, United Kingdom

**Corresponding Author:**

Prof. Attila Kardos, MD, FRCP, PhD, FESC,

Department of Cardiology, Translational Cardiovascular Research Group, Milton Keynes University Hospital NHS Foundation Trust, Milton Keynes, United Kingdom, 8H Standing Way, Eaglestone, Milton Keynes, MK6 5LD UK.

Email: [attila.kardos@cardiov.ox.ac.uk](mailto:attila.kardos@cardiov.ox.ac.uk)

***Dobutamines Stress echocardiography protocol***

Patients received patient information leaflets via the post and QR link to a patient information video to accustom themselves with the procedure at least a week prior the test. Patients received detailed explanation about the risks and benefit and possible side effects of the SE by the performing consultant cardiologist prior written consent on the day of the test. Patients were allowed to continue to take all their medication before SE. Patients were cannulated of their brachial vein and a three-way tap was connected for administering dobutamine, (atropine if required) and ultrasound enhancing agents (UEA, Sonovue or Luminity) via a rotator pump with set infusion rate with bolus injection setup (Bracco).

Vital signs including heart rate (HR), oxygen saturation, and monitor ECG were continuously recorded throughout the test and systolic and diastolic blood pressure (SBP, DBP) recoded on a 3-minute basis. Patients with inducible regional wall motion (RWM) abnormality (positive SE results) and / or who developed sustained arrythmia short acting betablockers (Esmolol) were given intravenously. Patients were observed for 30 minutes in the recovery phase before discharge.

Transthoracic echocardiograph (Philips EPIQ CVxi, X5-1 matrix transducer, Philips Medical Systems UK Limited) was used to assess the RWM of the left ventricular 17 segments at rest and during SE in five views: apical 4, 2 and 3 chamber views, and the parasternal long and short axis views at the papillary muscle level as per clinical recommendation (10,11). The RWM was recorded at each stage of the Dobutamine SE protocol (at rest, low dose Dobutamine, at peak/end of stress, and in the recovery). Quad images were recorded for offline analysis and to generate the wall motion score index for each stage. RWMA was assessed and scored for each individual 17 LV segments as normokinetic (1), hypokinetic (2), akinetic (3), dyskinetic (4) as the European and American Echocardiography guidelines (ref).

Dobutamine intravenous infusion in 4 stages (3 minutes each) started at 10mcg/kg/min in 10mcg/kg/min increment every three minutes to the maximum of 40mcg/kg/min was administered. In addition to dobutamine the protocol allowed the administration of intravenous Atropine from the beginning of stage 3 (150mcg bolus injection, to the maximum dose of 1200mcg), and "top up" handgrip exercise at the discretion of the performing cardiologist.

**Data extraction for QRISK3 cardiovascular risk calculation:**

QRISK3 variables were derived from natural language processing (NLP)-processed clinical text using a custom-built keyword dictionary developed iteratively from diagnoses, abbreviations, medications, and cardiovascular risk terminology. The extracted variables were mapped into structured QRISK3 inputs and used to calculate individual patient risk scores. Where data were incomplete or unavailable, predefined assumptions were applied to enable score generation. Specifically, missing hypertension treatment was assumed absent unless explicitly identified; diabetes status was classified as type 1, type 2, or absent based on detected terms; body mass index was estimated using average population height due to missing height data; ethnicity, cholesterol/HDL ratio, and systolic blood pressure variability were unavailable and excluded or defaulted; and the Townsend deprivation score was set to zero. The NLP extraction process incorporated extensive terminology relating to cardiovascular disease, hypertension, diabetes, smoking, atrial fibrillation, inflammatory disease, COPD/asthma, lipid disorders, and relevant pharmacological therapies to maximise identification of QRISK3-related variables from unstructured clinical records.
